# Supplementary material for: Crystal Structure Reveals the Full Ras–Raf Interface and Advances Mechanistic Understanding of Raf Activation
Source: Biomolecules. 2021 Jul 7;11(7):996. doi: 10.3390/biom11070996 (PMC8301913; doi:10.3390/biom11070996)
Supplement: Supplementary file 1 [file biomolecules-11-00996-s001.zip › biomolecules-1233311-supplementary.pdf]

Crystal structure reveals the full Ras:Raf interface and advances mechanistic understanding of Raf activation

Trinity Cookis[1] and Carla Mattos[1]*
[1]Department of Chemistry and Chemical Biology, Northeastern University, Boston, MA 02115, USA

*Correspondence: c.mattos@northeastern.edu

Supplementary Materials

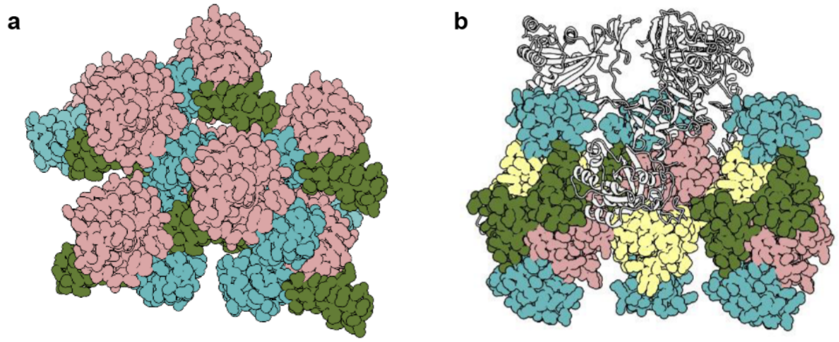

Figure S1. Crystal packing for HRas/CRaf-RBD_CRD and KRas/CRaf-RBD_CRD structures. (a) The HRas/CRaf-RBD_CRD (PDB ID 7JHP) crystal lattice does not contain the α4-α5 Ras dimer (or any other dimer). Ras is shown in pink, the Raf-RBD is shown in teal, and the Raf-CRD is shown in green. **(b)** The KRas/CRaf-RBD_CRD (PDB ID 6XI7) contains the α4-α5 Ras/Raf-RBD_CRD dimer, shown in vertical rows from this view of the crystal lattice: Ras (pink), Ras' (yellow), Raf-RBD (teal), Raf-CRD (green). The outer two layers show a view facing the two CRDs, which we propose is farthest from the membrane in cells (see Figure 3c for this membrane perspective). The middle row shows a view of the Ras surface that we propose is closest to the membrane. Molecules that make crystal contacts that are not part of the dimer are depicted as cartoon ribbon (white). KRas/CRaf-RBD_CRD coordinates (PDB ID 6XI7) were downloaded from the Protein Data Bank.

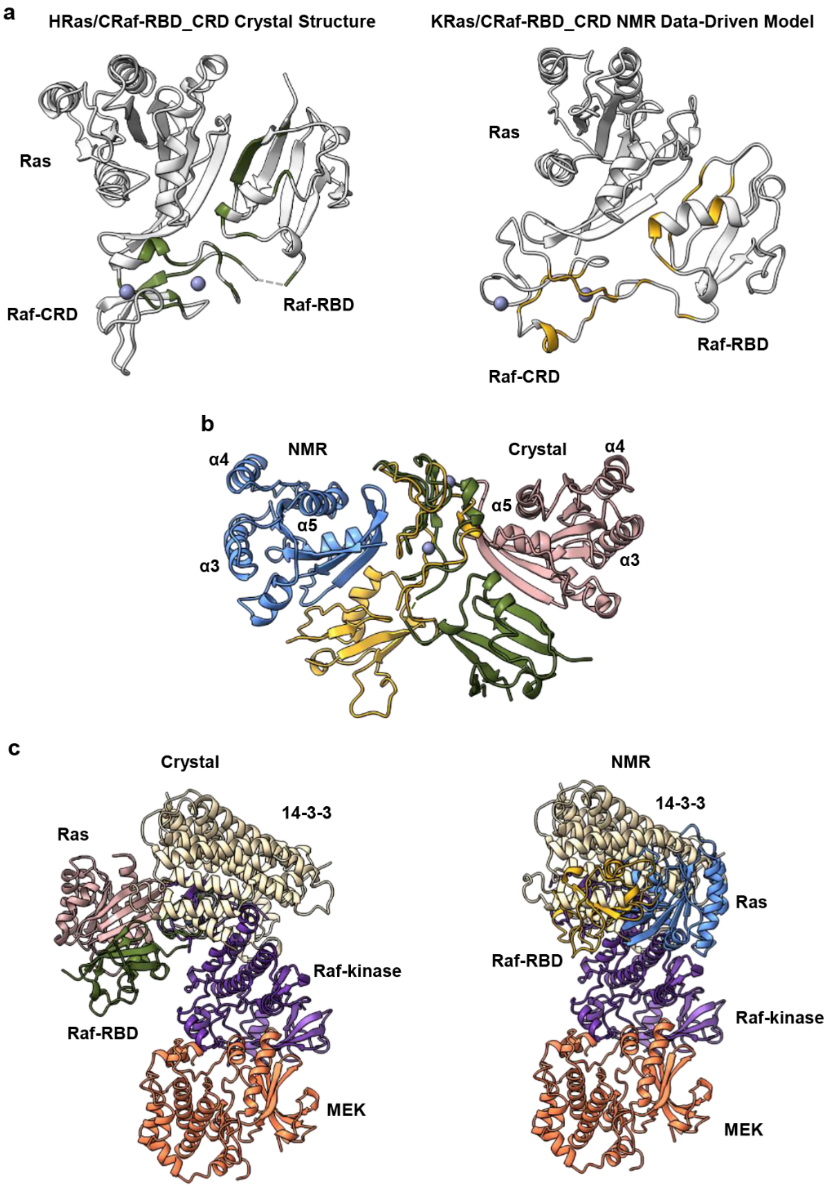

Figure S2. **Comparison of the HRas/CRaf-RBD_CRD crystal structure with nanodisc-bound KRas/CRaf-RBD_CRD NMR data-driven structure (a)** Chemical shift perturbations used to model the nanodisc-bound KRas/CRaf-RBD_CRD structure (PDB ID 6PTS) mapped onto our HRas/CRaf-RBD_CRD crystal structure (left, green) and the KRas/CRaf-RBD_CRD NMR-driven model (right, yellow). Our crystal structure is consistent with the NMR data used to determine the KRas/CRaf-RBD_CRD model. **(b)** Alignment of the Raf-CRDs from our crystal structure (Ras, pink; Raf-RBD_CRD, green) with that of the NMR model (Ras, blue; Raf-RBD_CRD, yellow) shows that the Raf-CRD is rotated 180˚ relative to Ras. **(c)** Comparison of our HRas/CRaf-RBD_CRD crystal structure (left) and the KRas/CRaf-RBD_CRD NMR-driven model (right) aligned with the BRaf-CRD from the inactive BRaf/14-3-3/MEK1 complex (PDB ID 6NYB). Only the Ras-binding interface identified in our crystal structure is accessible for Ras interaction in the autoinhibited state. Alignment of the Raf-CRDs between the NMR-driven model and autoinhibited structure results in the placement of the Raf-RBD in a position inconsistent with the cryo-EM data in which the Raf-RBD was solvent exposed and disordered.

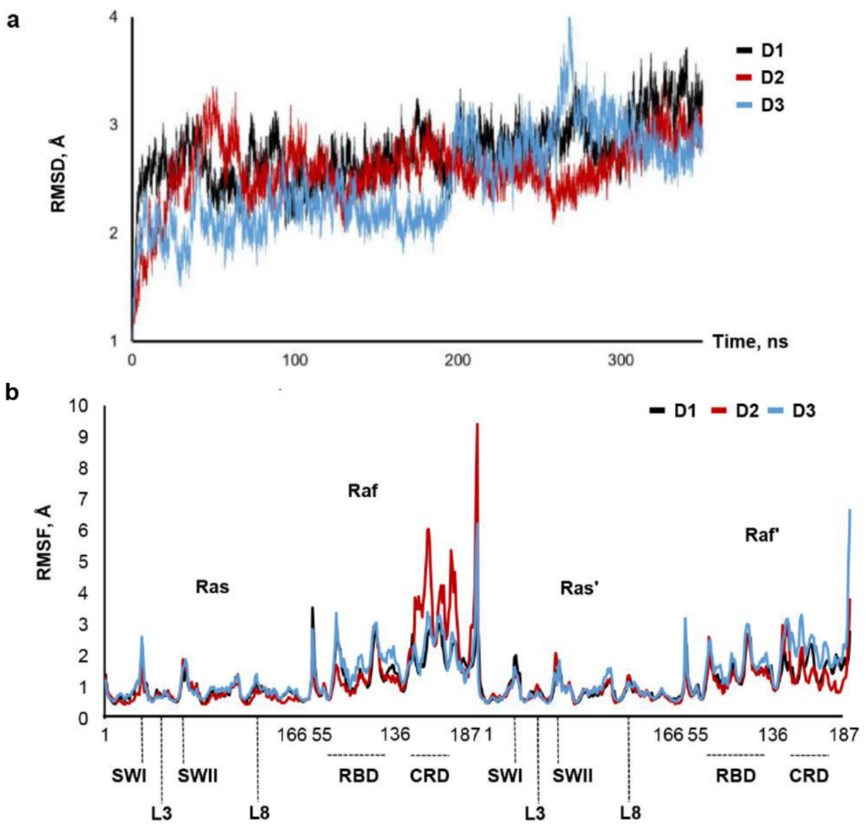

Figure S3. Molecular dynamics simulations of the dimer of the HRas/CRaf-RBD_CRD complex (a) Root-mean square deviation (RMSD) plots show that the three replicates of HRas/CRaf-RBD_CRD dimer simulations (D1, D2, D3) converge and are stable over 350 ns of simulation time. **(b)** Root-mean square fluctuations (RMSF) analyses for the dimer simulations (D1, D2, D3) show enhanced fluctuations in the Raf-CRD that vary among replicates consistent with the dynamic variability of Ras/Raf-CRD interactions at loop 3 and loop 8 involving both Ras protomers.

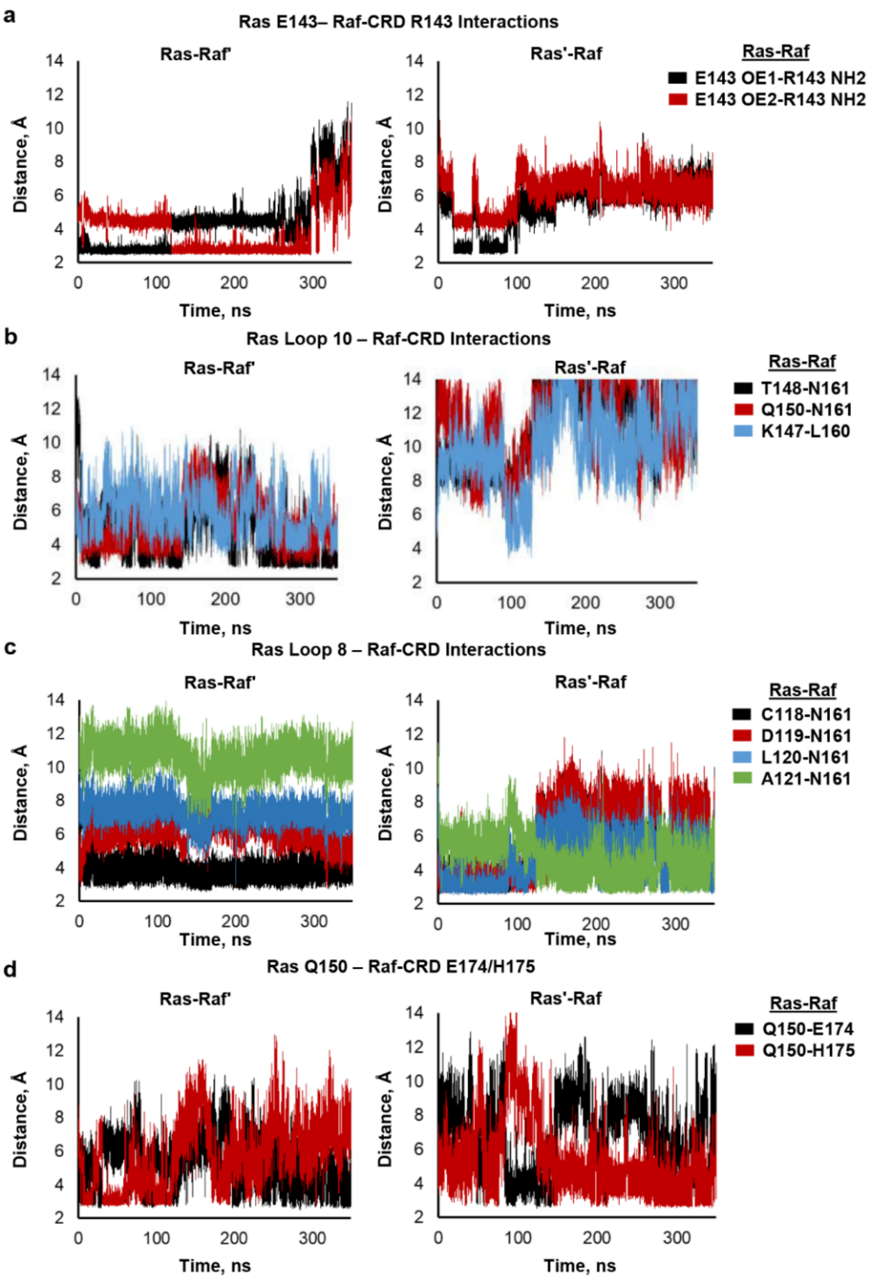

Figure S4. Ras/Raf-CRD' and Ras'/Raf-CRD interactions over 350 ns simulation time of the HRas/CRaf-RBD_CRD dimer complex (replicate 1). (a) Distances calculated between the sidechain of Raf-CRD residue R143 and the side chain of residue E143 of the opposing Ras protomer. Ras/Raf-CRD' interactions are shown on the left and Ras'/Raf-CRD interactions are shown on the right. **(b)** Distances calculated between the sidechain of Raf-CRD residue N161 and the side chains of K147 (blue), T148 (black), and Q150 (red) located in loop 10 of the opposing Ras protomer. **(c)** Distances calculated between the sidechain of Raf-CRD residue N161 and the carbonyl backbone of residues C118 (black), D119 (red), L120 (blue), and A121 (green) in the opposing Ras molecule. **(d)** Distances calculated between the sidechains of Raf-CRD residues E174 (black) and H175 (red) and the side chain of residue Q150 of the opposing Ras protomer.

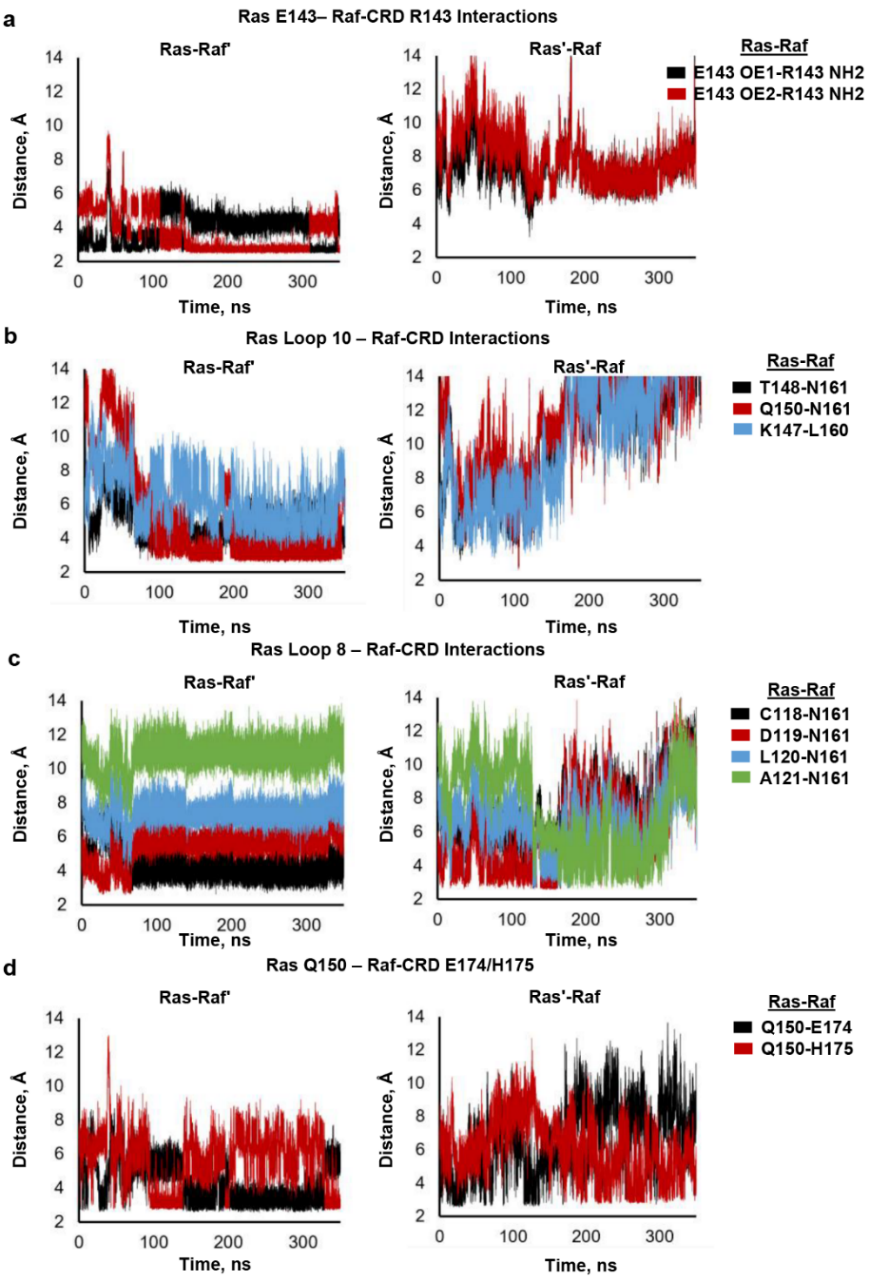

Figure S5. Ras/Raf-CRD' and Ras'/Raf-CRD interactions over 350 ns simulation time of the HRas/CRaf-RBD_CRD dimer complex (replicate 2). (a) Distances calculated between the sidechain of Raf-CRD residue R143 and the side chain of residue E143 of the opposing Ras protomer. Ras/Raf-CRD' interactions are shown on the left and Ras'/Raf-CRD interactions are shown on the right. **(b)** Distances calculated between the sidechain of Raf-CRD residue N161 and the side chains of K147 (blue), T148 (black) and Q150 (red) located in loop 10 of the opposing Ras protomer. **(c)** Distances calculated between the sidechain of Raf-CRD residue N161 and the carbonyl backbone of residues C118 (black), D119 (red), L120 (blue), and A121 (green) in the opposing Ras molecule. **(d)** Distances calculated between the sidechains of Raf-CRD residues E174 (black) and H175 (red) and the side chain of residue Q150 of the opposing Ras protomer.

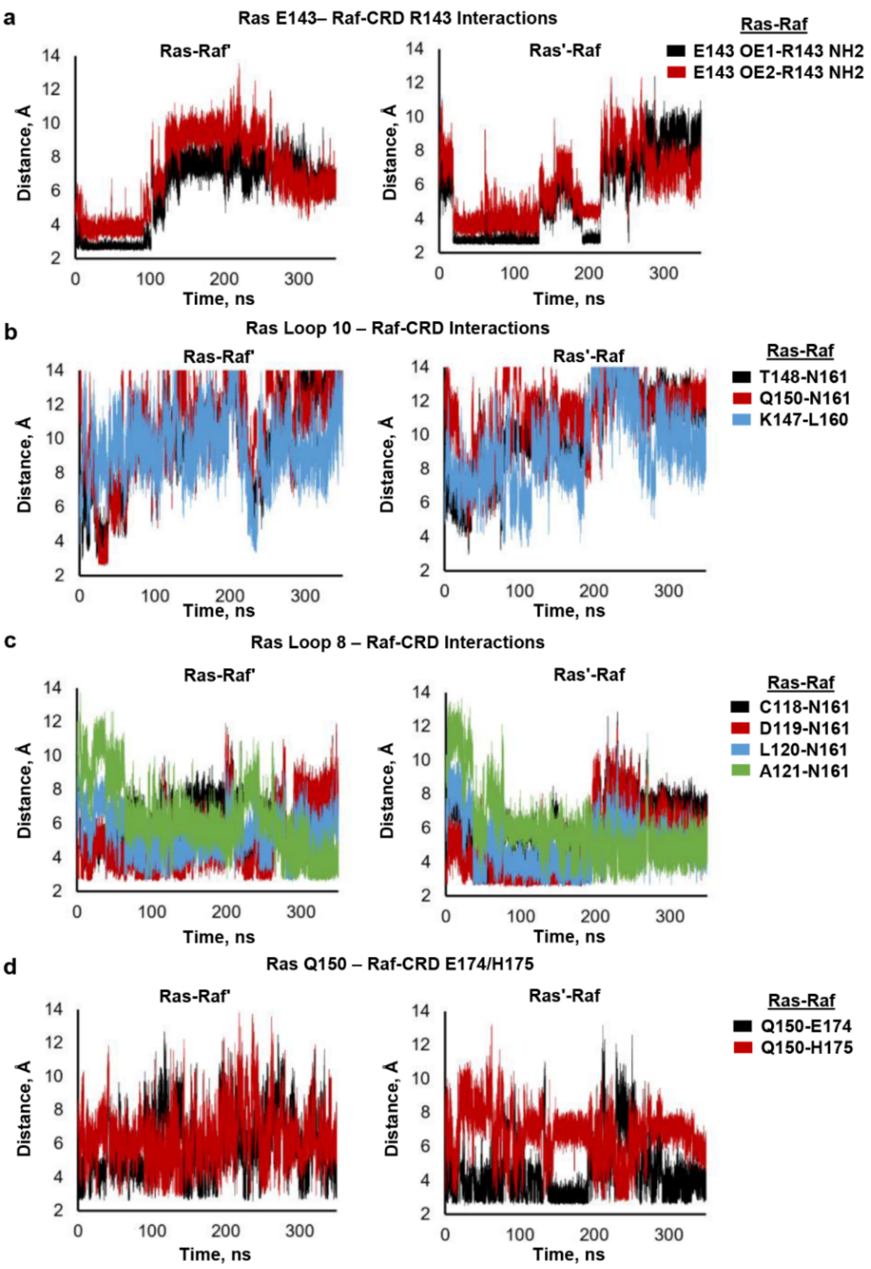

Figure S6. Ras/Raf-CRD' and Ras'/Raf-CRD interactions over 350 ns simulation time of the HRas/CRaf-RBD_CRD dimer complex (replicate 3). (a) Distances calculated between the sidechain of Raf-CRD residue R143 and the side chain of residue E143 of the opposing Ras protomer. Ras/Raf-CRD' interactions are shown on the left and Ras'/Raf-CRD interactions are shown on the right. **(b)** Distances calculated between the sidechain of Raf-CRD residue N161 and the side chains of K147 (blue), T148 (black) and Q150 (red) located in loop 10 of the opposing Ras protomer. **(c)** Distances calculated between the sidechain of Raf-CRD residue N161 and the carbonyl backbone of residues C118 (black), D119 (red), L120 (blue), and A121 (green) in the opposing Ras molecule. **(d)** Distances calculated between the sidechains of Raf-CRD residues E174 (black) and H175 (red) and the side chain of residue Q150 of the opposing Ras protomer.

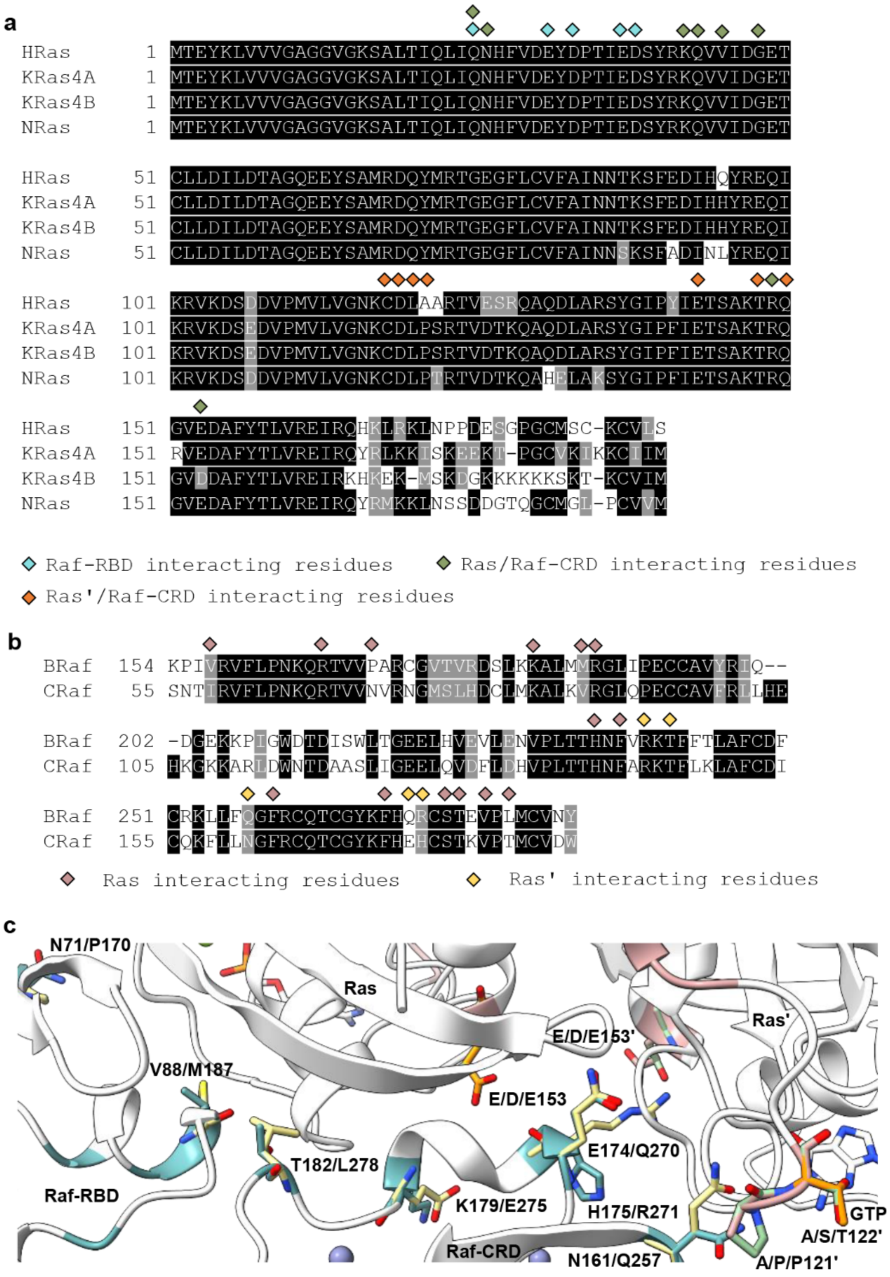

Figure S7. Ras and Raf isoform specific residues at the Ras/Raf interface. (a) Sequence alignment of the four Ras isoforms (HRas, KRas4A, KRas4B, and NRas) with residues denoted with a diamond corresponding to those that interact with the Raf-RBD (blue), with the Raf-CRD through the loop 3 interface (green), and with the Raf-CRD through the loop 8 interface (orange). **(b)** Sequence alignment of BRaf and CRaf conserved region 1 containing the Raf-RBD and CRD. Residues denoted with a pink diamond are involved in interactions at the Ras loop 3 / Raf-CRD interface and residues denoted with a yellow diamond are involved in interactions at the Ras' loop 8 / Raf-CRD interface. **(c)** Close-up view of the Ras/Raf-CRD interfaces with isoform specific residue sidechains shown for CRaf (blue), BRaf (yellow), HRas (pink), KRas (green), and NRas (orange).

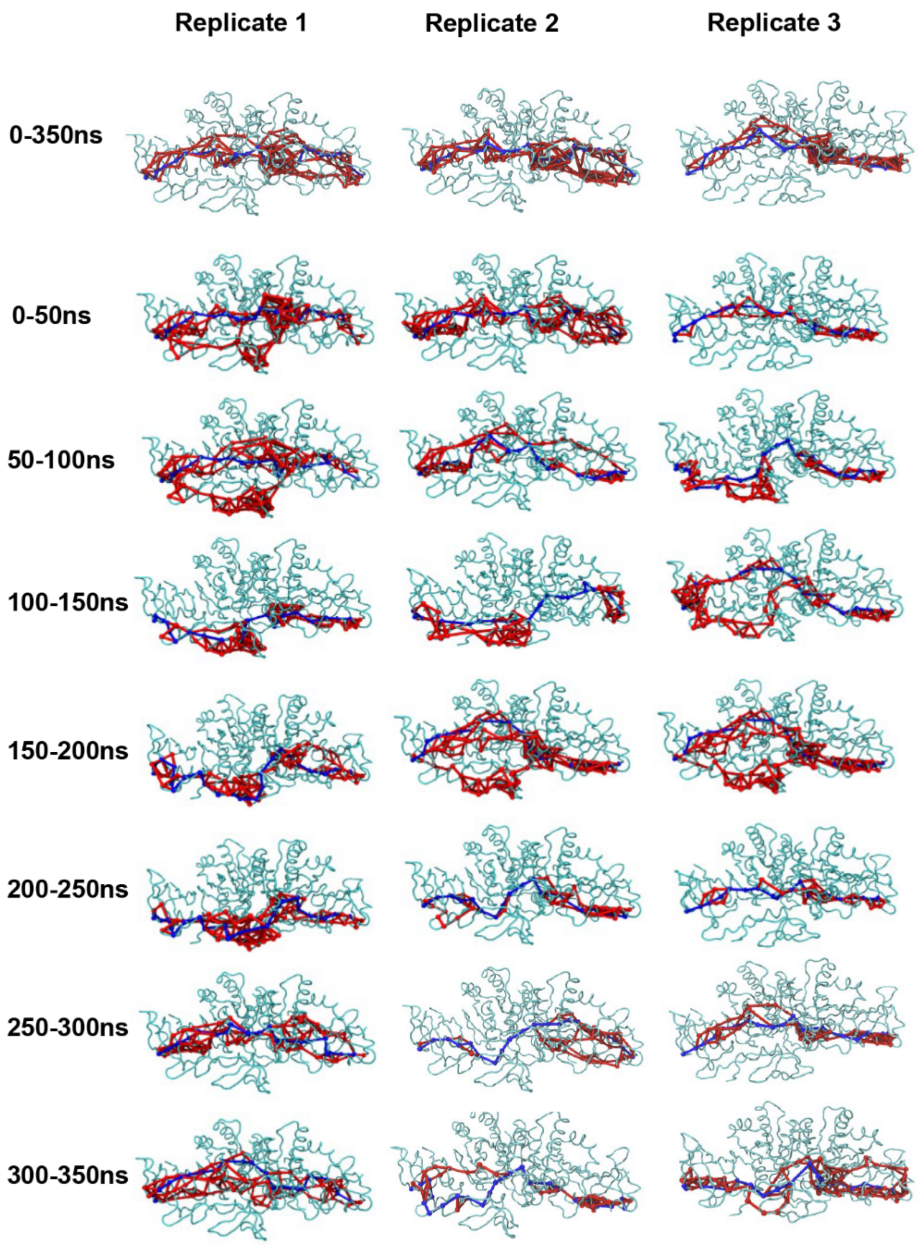

Figure S8. Network view of all optimal and suboptimal path calculations between Raf-RBD residues D113 and D113'. Visualization of paths calculated for the entire 350 ns MD trajectories and the smaller 50 ns segments for the three replicates of the HRas/CRaf-RBD_CRD model showing the 4 modes of intermolecular information transfer involving helix 5, loop 3, and loop 8 regions of the Ras G-domain and the Raf-CRD. The optimal path, which corresponds to that with the least number of nodes, is shown in blue. All other paths are designated as suboptimal paths and are shown in red.

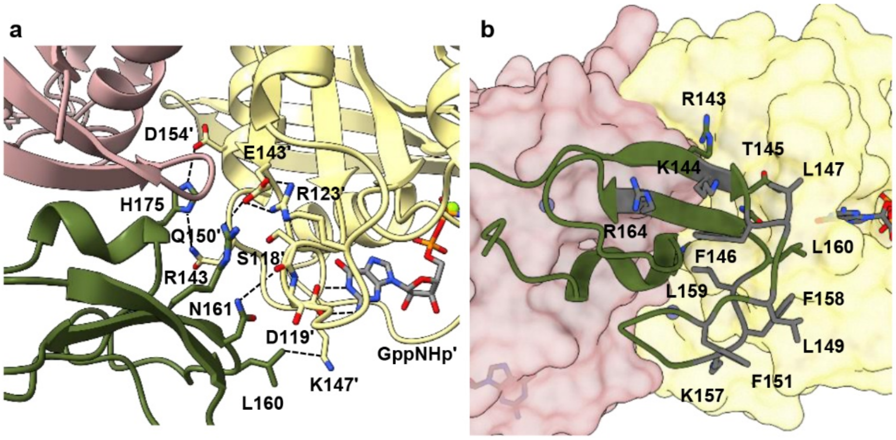

Figure S9. KRas/CRaf-RBD_CRD dimer in the crystal (PDB ID 6XI7). (a) Close-up view of Raf-CRD (green) interactions with Ras' (yellow) in the KRas/CRaf-RBD_CRD dimer generated by a 2-fold crystallographic symmetry rotation applied to the asymmetric unit. **(b)** Proposed membrane-binding residues (grey) in the Raf-CRD (green) form a layer of hydrophobic interactions at the Raf-CRD core that present residues R143, T145, L160, N161, and H175 for interaction with Ras' (yellow) in the KRas/CRaf-RBD_CRD complex. The Ras protomer making the primary interactions with the Raf-CRD in the asymmetric unity (pink) is shown in both panels for context. The KRas/CRaf-RBD_CRD coordinates were downloaded from the Protein Data Bank (PDB ID 6XI7).

Table S1. X-Ray data collection and structure refinement statistics

HRas/CRaf-RBD_CRD complex (PDB 7JHP)	
**Data Collection**	
Space group	C 1 2 1
Unit cell dimensions	
a, b, c (Å)	94.49, 44.35, 74.40
α, β, γ (°)	90, 103.95, 90
Resolution (Å)	36.61-2.77 (2.87-2.77)
I/σ	15.63 (2.08)
Completeness (%)	91.48 (49.27)
Redundancy	7.2 (6.9)
Wilson B-factor	45.00
R_{merge}	0.120 (0.785)
R_{meas}	0.126 (0.818)
CC1/2	(0.795)
**Refinement**	
No. reflections	349414
Unique reflections	7158 (369)
R_{work}	0.2107 (0.2641)
R_{free}	0.2633 (0.3404)
No. atoms	2311
Macromolecules	2228
Ligands	35
Solvent	48
RMS (bonds)	0.002
RMS (angles)	0.45
Ramachandran (%)	
favored	96.48
allowed	3.17
outliers	0.35
Average B-factor	50.88
Macromolecules	51.25
Ligands	40.16
Solvent	41.39

*Values in parentheses are for highest-resolution shell.

Table S2. Raw edge weights, d_{ij}, calculated between residues located at the Ras/Raf-CRD' interface from global dynamical network analysis of each 350 ns MD simulation of the HRas/CRaf-RBD_CRD dimer.

Residue Pair	Replicate 1 d_{ij}	Replicate 2 d_{ij}	Replicate 2 d_{ij}
C118-N161	1.83	1.39	-
D119-T145	1.39	1.51	-
D119-N161	1.51	1.38	-
A120-T145	1.38	-	-
A121-T145	1.64	1.64	1.86
R123-R143	2.29	2.29	-
E143-R143	2.55	-	-
T148-L160	1.55	-	-
Q150-N161	1.60	-	-
C118-R143	2.01	1.83	
Q150-E174	2.41	2.01	1.55
Q150-H175	-	2.41	1.86

*Edge weights are calculated as a function of residue pair-wise cross correlation with the relationship: $d_{ij} = -\log(|C_{ij}|)$.

Table S3. Raw edge weights, d_{ij}, calculated between residues located at the Ras/Raf-RBD_CRD interface from global dynamical network analysis of each 350 ns MD simulation of the HRas/CRaf-RBD_CRD dimer.

Residue Pair	Replicate 1 D_{ij}	Replicate 2 d_{ij}	Replicate 2 d_{ij}
I21-V88	0.92	1.01	0.57
I24-V88	0.84	0.77	0.45
Q25-K87	1.17	1.27	0.66
Q25-V88	0.89	0.88	0.44
E31-K84	1.80	-	-
E33-K84	1.26	1.53	1.73
I36-T57	4.04	-	1.55
I36-V69	0.83	0.67	0.83
I36-N71	-	1.17	1.62
E37-R59	1.22	-	-
E37-R67	0.50	0.57	0.88
E37-T68	0.45	0.46	0.73
E37-V69	0.77	0.49	0.83
D38-R67	0.34	0.34	0.65
D38-T68	0.31	0.30	0.52
D38-S89	0.67	0.85	0.70
S39-Q66	0.31	0.27	0.65
S39-R67	0.30	0.24	0.70
S39-S89	0.61	0.64	0.77
Y40-Q66	0.39	0.30	0.78

Y40-V88	0.63	0.54	0.56
Y40-S89	0.49	0.49	0.49
R41-N64	0.61	0.44	1.04
R41-K65	0.61	0.40	1.09
R41-Q66	0.49	0.32	1.02
K42-T178	1.80	3.52	1.08
Q43-N140	3.03	-	-
Q43-F141	3.53	-	0.89
Q43-S177	-	-	0.86
Q43-H139	-	-	1.16
V44-S177	-	-	0.68
V44-T178	0.93	1.93	0.81
V45-F141	-	-	1.24
V45-F163	1.49	-	1.06
V45-T178	-	2.04	-
V45-E174	0.71	2.44	0.52
T50-S177	-	-	1.20
T50-F163	1.78	-	
L56-R67	0.63	0.46	-
R149-K179	-	1.91	-
Y157-T178	-	2.06	-
N26-K179	-	0.77	3.54

*Edge weights are calculated as a function of residue pair-wise cross correlation with the relationship: $d_{ij} = -\log(|C_{ij}|)$.

Table S4. Raw edge weights, d_{ij}, calculated between residues located at the Ras/Ras' interface (dimerization interface) from global dynamical network analysis of entire 350 ns HRas/CRaf-RBD_CRD MD simulations.

Residue Pair	Replicate 1 D_{ij}	Replicate 2 d_{ij}	Replicate 2 d_{ij}
Q127-D47	1.15	-	0.83
Q127-G48	0.68	-	1.09
V128-E49	1.03	-	-
L131-D47	1.68	-	-
L131-E49	1.04	-	1.06
L131-R164	1.27	0.90	1.04
R135-R164	0.99	0.75	1.10
R135-Q165	0.78	0.74	0.96
R135-H166	0.65	1.04	1.24
G138-Q165	0.74	0.81	1.30
I139-Q165	0.82	0.80	1.25
P140-Q165	1.13	-	1.32
Y141-D47	1.84	2.16	0.58
Y141-R161	1.78	1.99	0.98
I142-D47	1.52	1.77	0.40
I142-R161	2.09	1.38	0.91
E143-D47	-	1.62	0.41
E143-G48	-	1.82	0.59
D154-D154	2.04	1.29	1.15
R161-Y141	0.56	0.47	0.88

R161-I142	0.62	0.63	0.81
E162-E162	1.21	1.65	-
I163-R135	-	0.72	-
R164-Q131	0.58	0.65	0.91
R164-R135	0.55	0.59	0.89
Q165-R135	0.60	0.58	0.86
Q165-S136	0.75	-	-
Q165-Y137	0.72	-	-
Q165-G138	0.75	0.61	1.27
Q165-I139	-	0.52	1.41
Q165-P140	-	0.58	1.57
Q165-E162	-	2.46	-
H166-R135	0.57	0.83	1.25
Y4-Q131	1.30	1.20	-
D47-S127	0.46	0.86	1.07
D47-Y141	0.56	0.35	0.74
D47-I142	0.48	0.30	0.59
D47-E143	0.63	0.31	0.58
G48-S127	0.41	0.87	1.55
G48-E143	0.81	0.43	0.87
E49-Q131	0.55	-	1.30
E49-S127	-	0.78	-

*Edge weights are calculated as a function of residue pair-wise cross correlation with the relationship: $d_{ij} = -\log(|C_{ij}|)$.

Table S5. Residues involved in intermolecular information transfer at the Ras dimerization interface identified in optimal/suboptimal path calculations between Raf-RBD residues D113-D113' and D117-D117' (replicate 1).

1	D113-D113' paths	D117-D117' paths
**0-50 ns**	465 paths<br>Ras G48-Ras' S127 80.4%<br>Raf N161 – Ras' D119/L120 13.9%<br>Ras D47-Ras' I142 2.9%<br>Raf S177-Ras V45 2.4%	652 paths<br>Ras D47/G48-Ras' S127 92.3%<br>Ras D47-Ras' I142/E143 6.9%<br>Ras R161-Ras' I142 0.3%
**50-100 ns**	864 paths<br>Raf L160/N161-Ras' D119 60.7%<br>Ras D47/G48-Ras' I142/E143 35.4%	116 paths<br>Ras D47/G48-Ras' I142/E143 89.6%<br>Ras G48-Ras' S127 8.6%
**100-150 ns**	2209 paths<br>Raf E174 – Ras' Q150 100%	8369 paths<br>Raf E174 – Ras' Q150 100%
**150-200 ns**	1902 paths<br>Ras C118 - Raf' N161 72.4%<br>Ras D119 - Raf' N161 24.6%	1438 paths<br>Ras C118 - Raf' N161 71.3%<br>Ras D119 - Raf' N161 28.7%
**200-250 ns**	1923 paths<br>Ras C118/D119 - Raf' L160/N161 58.2%<br>Ras C118/D119 - Raf' T145/F146 35.7%<br>Ras T148 - Raf' L160 6.1%	6878 paths<br>Ras C118/D119 - Raf' L160/N161 60.4%<br>Ras C118/D119 - Raf' T145/F146 34.1%<br>Ras T148 - Raf' L160 5.4%
**250-300 ns**	1923 paths<br>Ras D47 - Ras' I142/E143 100%	6878 paths<br>Ras D47 - Ras' I142/E143 100%
**300-350 ns**	265 paths<br>Ras R161 - Ras' I142 68.3%<br>Ras T148 - Raf' N161 16.6%<br>Ras D47/G48 - Ras' I142/E143 15.1%	78 paths<br>Ras R161 - Ras' I142 75.6%<br>Ras D47/G48 - Ras' I142/E143 23.4%
**0-350 ns**	1500 paths<br>Ras D47 - Ras' I142/E143 98.5%<br>Ras R161 – Ras' I142 1.5%	502 paths<br>Ras D47 - Ras' I142/E143 84.3%<br>Ras R161 – Ras' I142 15.7%

*Percentages were calculated as the number of times each edge appeared in the suboptimal path calculations divided by the total number of calculated paths.

Table S6. Residues involved in intermolecular information transfer at the Ras dimerization interface identified in optimal/suboptimal path calculations between Raf-RBD residues D113-D113' and D117-D117' (replicate 2).

2	D113-D113' paths	D117-D117' paths
**0-50ns**	1871 paths<br>Ras D47-Ras' I142/E143 100%	1195 paths<br>Ras D47-Ras' I142/E143 99.4%
**50-100 ns**	313 paths<br>Ras D47-Ras' I142 87.5%<br>Ras R161-Ras' I142 12.5%	1110 paths<br>Ras D47-Ras' I142/E143 92.2%<br>Ras R161-Ras' I142 7.8%
**100-150 ns**	155 paths<br>Ras C118-Raf' N161 100%	111 paths<br>Ras C118-Raf' N161 100%
**150-200 ns**	9897 paths<br>Ras R161-Ras' I142 90.6%<br>Ras D47-Raf' I142/E143 8.5%<br>Raf N161-Ras' 121 0.8%	2099 paths<br>Ras R161-Ras' I142 97.0%<br>Ras D47-Raf' I142/E143 2.4%
**200-250 ns**	78 paths<br>Raf T178-Ras V45 93.5%<br>Ras D47-Ras‘ I142 100%	96 paths<br>Raf T178-Ras V45 94.7%<br>Ras D47-Ras' I142 99.0%
**250-300 ns**	108 paths<br>Raf T178-Ras V45 100%<br>Ras D47-Ras' I142 100%	49 paths<br>Raf T178-Ras V45 100%<br>Ras D47-Ras' I142 100%
**300-350 ns**	99 paths<br>Raf T178-Ras V45 98%<br>Ras D47-Ras' I142 100%	31 paths<br>Raf T178-Ras V45 100%<br>Ras D47-Ras' I142 100%
**0-350 ns**	4513 paths<br>Ras D47/G48-Ras' I142/E143 100%	1327 paths<br>Ras D47/G48-Ras' I142/E143 100%

*Percentages were calculated as the number of times each edge appeared in the suboptimal path calculations divided by the total number of calculated paths.

Table S7. Residues involved in intermolecular information transfer at the Ras dimerization interface identified in optimal/suboptimal path calculations between Raf-RBD residues D113-D113' and D117-D117' (replicate 3).

3	D113-D113' paths	D117-D117' paths
**0-50ns**	87 paths<br>Ras D154-Ras' D154 97.7%	230 paths<br>Ras I142/E143-Ras' D47 55.6%<br>Ras D154-Ras' D154 42.4%
**50-100 ns**	1083 paths<br>Raf E174-Ras V45 45.8%<br>Raf S177-Ras V45 36.6%<br>Raf F163-Ras V45 17.6%<br>Ras D47-Ras' I142 100%	1839 paths<br>Raf E174-Ras V45 42.9%<br>Raf S177-Ras V45 38.8%<br>Raf F163-Ras V45 14.7%<br>Ras D47-Ras' I142 99.9%
**100-150 ns**	420 paths<br>Ras R161-Ras' I142 78.3%<br>Ras D47-Raf' I142/E143 21.2%	147 paths<br>Ras R161-Ras' I142 100%
**150-200 ns**	8692 paths<br>Ras L120-Raf' R143 87.0%<br>Ras D119-Raf' R143 10.8%<br>Ras C118-Raf' R143 1.5%<br>Ras A121-Raf'143 0.6%	10260 paths<br>Ras L120-Raf' R143 84.0%<br>Ras D119-Raf' R143 12.3%<br>Ras C118-Raf' R143 3.0%<br>Ras A121-Raf'143 0.7%
**200-250 ns**	151 paths<br>Ras I142/E143-Ras' D47 100%	695 paths<br>Ras I142-Ras' R161 56.7%<br>Ras I142-Ras' D47 43.2%
**250-300 ns**	755 paths<br>Ras I142/E143-Ras' D47 99.5%<br>Ras I142-Ras′ R161 0.5%	741 paths<br>Ras I142/E143-Ras' D47 99.5%<br>Ras I142-Ras′ R161 0.5%
**300-350 ns**	3073 paths<br>Ras E143-Raf′ E174 76.6%<br>Ras I142-Ras′ D47 23.4%<br>Ras′ D47-Raf′ E174 19.0%<br>Ras′ D47-Raf′ T178 4.4%	183 paths<br>Ras E143-Raf′ E174 97.3%<br>Ras′ V45-Raf′ E174 2.7%<br>Ras I142/E143-Ras′ D47 2.7%
**0-350 ns**	7944 paths<br>Ras I142/E143-Ras' D47 97.8%<br>Ras D154-Ras′ D154 1.1%<br>Ras I142-Ras′ R161 1.1%	3060 paths<br>Ras I142-Ras′ R161 69.9%<br>Ras I142/E143-Ras′ D47 27.9%<br>Ras D154-Ras′ D154 2.2%

*Percentages were calculated as the number of times each edge appeared in the suboptimal path calculations divided by the total number of calculated paths.
